# A Distinct Expression Pattern of Cyclin K in Mammalian Testes Suggests a Functional Role in Spermatogenesis

**DOI:** 10.1371/journal.pone.0101539

**Published:** 2014-07-08

**Authors:** Xiaocong Xiang, Li Deng, Jingli Zhang, Xudong Zhang, Tingjun Lei, Guangxin Luan, Chunlei Yang, Zhi-Xiong Xiao, Qian Li, Qintong Li

**Affiliations:** 1 Center of Growth, Metabolism, and Aging, Key Laboratory of Bio-Resources and Eco-Environment, and State Key Laboratory of Biotherapy, College of Life Sciences, Sichuan University, Chengdu, Sichuan, China; 2 363 Hospital, Aviation Industry Corporation of China, Chengdu, Sichuan, China; University Hospital of Münster, Germany

## Abstract

Germ cell and embryonic stem cells are inextricably linked in many aspects. Remarkably both can generate all somatic cell types in organisms. Yet the molecular regulation accounting for these similarities is not fully understood. Cyclin K was previously thought to associate with CDK9 to regulate gene expression. However, we and others have recently shown that its cognate interacting partners are CDK12 and CDK13 in mammalian cells. We further demonstrated that cyclin K is essential for embryonic stem cell maintenance. In this study, we examined the expression of cyclin K in various murine and human tissues. We found that cyclin K is highly expressed in mammalian testes in a developmentally regulated manner. During neonatal spermatogenesis, cyclin K is highly expressed in gonocytes and spermatogonial stem cells. In adult testes, cyclin K can be detected in spermatogonial stem cells but is absent in differentiating spermatogonia, spermatids and spermatozoa. Interestingly, the strongest expression of cyclin K is detected in primary spermatocytes. In addition, we found that cyclin K is highly expressed in human testicular cancers. Knockdown of cyclin K in a testicular cancer cell line markedly reduces cell proliferation. Collectively, we suggest that cyclin K may be a novel molecular link between germ cell development, cancer development and embryonic stem cell maintenance.

## Introduction

It has long been recognized that embryonic stem cells (ESCs) and germ cells share important similarities. Both are pluripotent, that is, they can generate all somatic cell types in organisms. Underlying this remarkable ability is inextricably linked molecular regulatory networks that when dysregulated can lead to human diseases such as cancer [1]. This is exemplified by facts that discoveries of ESCs were based on investigations on testicular tumors [2], primordial germ cells (PGCs) can form ESC-like cells in culture [3], and ectopic expression of a subset of germline genes, termed cancer/testis antigens, is frequently observed in human cancers [4]. Thus understanding the molecular circuitries that regulate these similarities will certainly have implications in regenerative medicine and cancer therapies [5].

Spermatogenesis is the process by which sperm cells or spermatozoa are produced from PGCs in the seminiferous tubules of the testes [6]–[8]. It begins as gonocytes, derived from PGCs, migrate from the center of seminiferous tubules to the basal membrane [9]. Once attached to the basal membrane, gonocytes transform into spermatogonial stem cells (SSCs) or A_single_ (As) cells between 0 and 6 days postpartum (dpp) in mice [10]. A portion of SSCs self-renew throughout life, while others take on the differentiation path to generate progenitor cells through mitosis (termed A_paired_ and A_aligned_ cells containing two to sixteen cells connected by cytoplasmic bridges due to lack of complete cytokinesis), and then spermatocytes through meiosis. Spermatocytes further mature into spermatids and finally spermatozoa [6], [7]. Successful spermatogenesis depends on the microenvironment in the seminiferous tubules formed by somatic cells termed Sertoli cells. In addition, Leydig cells and myoid cells in interstitial tissues may also contribute to spermatogenesis microenvironment [8].

Intriguingly, many pluripotency genes, highly expressed in ESCs, are also detected in germ cells at different developmental stages although their functions in spermatogenesis are less understood [1]. Core pluripotency transcription factors Oct4 and Sall4 of ESCs are highly expressed in PGCs, gonocytes and SSCs but not in their differentiated derivatives [11], [12]. In contrast, key pluripotency transcription factor Nanog of ESCs is highly expressed in PGCs and gonocytes but not in SSCs. Instead, it is specifically expressed in spermatocytes in adult testes [13], [14]. Therefore it seems that although core pluripotency transcription factors collaborate to maintain self-renewal in ESCs [15], [16], they participate in different cellular processes during spermatogenesis. Nevertheless, the unbroken cycle of pluripotency between early epiblast and germline is sustained by inextricably linked molecular regulatory networks [1].

The physiological functions of cyclin K are poorly understood. The important role of cyclin K is manifested by the fact that genetic ablation in mice leads to embryonic lethality before the blastocyst stage [17]. Recombinant cyclin K protein was previously shown to interact with CDK9 protein in vitro [18], and thought to function as positive transcription elongation factor b (P-TEFb) to regulate RNA polymerase II transcription elongation [19]. However, several labs have recently demonstrated that in cells cyclin K interact with CDK12 and CDK13 instead of CDK9 [17], [20], [21]. Thus the function of cyclin K needs to be revaluated in this new context. We further showed that cyclin K is required to maintain self-renewal in murine ESCs, offering a potential explanation to the lethal phenotype during early embryogenesis in knockout studies [21]. Current knowledge of CDK12 and CDK13 is also scarce although there is evidence suggesting that they may be involved in transcription and/or RNA processing [22]–[24].

In this study, we started to explore the function of cyclin K under physiological and diseased conditions. We found that cyclin K is highly expressed in mammalian testes in a developmentally regulated manner. During neonatal spermatogenesis, cyclin K is highly expressed in gonocytes and SSCs. In adult testes, the most prominent expression is localized in spermatocytes. Furthermore, cyclin K is highly expressed in human testicular tumors and seems to be required for tumor cell proliferation. Taken together, we suggest that cyclin K may be a novel molecular link between germ cell development, human cancer development and embryonic stem cell maintenance.

## Materials and Methods

### Cell Culture and Tissue Samples

Embryonal carcinoma cell line F9 was obtained from American Type Culture Collection (ATCC) and is cultured in Dulbecco Modified Eagle Medium (DMEM) containing 10% Fetal Bovine Serum (FBS). Culture plate is coated with 0.1% gelatin prior to use. Neonatal and adult tissues from BALB/c mice were dissected, fixed immediately in 10% formalin/PBS (137 mM NaCl, 2.7 mM KCl, 10 mM Na_2_HPO_4_, 2 mM KH_2_PO_4_) overnight, dehydrated stepwise by 70%, 80%, 90%, 95% and 100% ethanol, cleared in xylene and embedded in paraffin. All procedures involving mice were performed in accordance with the Guide for the Care and Use of Laboratory Animals approved by the Institutional Animal Care and Use Committee of Sichuan University. Normal and diseased human tissues were obtained from the AVIC 363 Hospital. Written informed consent from donors or the next of kin was obtained for use of these samples in research, and was approved by the Ethical Committee of the AVIC 363 Hospital. The data were analyzed anonymously.

### Lentivirus Infection

F9 cells were transduced with lentivirus containing individual pLKO.1 shRNA construct [21] for two days, then split equally into three wells on 6-well plates (10^5^ cells per well) in growth medium supplemented with 2.5 µg/ml puromycin (Sigma) and grown for another three days. One well was stained by crystal violet, one for protein blot analysis, and one for counting cell number.

### Protein Extraction and Western Blot Analysis

Mouse tissues were collected from 2-month-old BALB/c mice, ground in liquid nitrogen with a mortar and pestle, and transferred to a 1.5 ml Eppendorf tube with pre-cooled extraction buffer (50 mM HEPES, pH 7.5, 140 mM NaCl, 1 mM EDTA pH 8.0, 1% Triton X-100, 0.1% sodium deoxycholate, 0.1% SDS, proteinase inhibitors (Roche), 0.1% PMSF). Lysates were incubated on ice for 1 h, vortexed every 10 min and cleared at 17,000 g/4°C for 15 min. Cleared cell lysates were separated by 10% SDS-PAGE, followed by protein blot analyses. Anti-cyclin K antibody was previously described [21]. Anti-Gapdh antibody was purchased from Kangchen Biotech (China).

### RNA Extraction and qPCR Analysis

Total RNA was extracted by TRIzol (Invitrogen) following the manufacturer's instructions, treated with DNase I (Ambion), and reverse-transcribed by random hexamers using SuperScript II (Invitrogen). Quantitative PCR (qPCR) was performed by using EvaGreen (Bio-Rad) on an iCycler Thermal Cycler (Bio-Rad). Following primers are used: cyclin K-F: GTTACACTATGACACCCTGGC and cyclin K-R: GTTTCCTCTACTTTCCCAGCC, Gapdh-F: TGCACCACCAACTGCTTAGC and Gapdh-R: GGCATGGACTGTGGTCATGAG. The amount of cyclin K mRNA was normalized and quantitated to that of Gapdh and quantified as previously described [25].

### Immunohistochemistry

Paraffin-embedded samples were sectioned into 3 µm-thick sections and mounted on positively charged slides. Sections were dewaxed two times in xylene, rehydrated with 100%, 90%, 70%, and 50% ethanol, subjected to antigen retrieval in 10 mM citrate buffer (pH 6.0) in a pressure cooker for 3 min and allowed to cool to room temperature, and then rinsed in PBS for 10 min. Endogenous peroxidases were quenched by incubation with 3% H_2_O_2_ for 10 min. Sections were permeated with 0.1% Triton X-100 in PBS for 10 min, washed twice with PBS, and blocked with 3% bovine serum albumin in PBS for 1 h at room temperature. Sections were then incubated overnight at 4°C with primary antibodies, washed in PBS four times, and then incubated with the horseradish peroxidase-conjugated anti-rabbit antibody (pv-6001, ZSGQ-BIO, China) for 60 min at room temperature. After washing in PBS four times, sections were visualized by 3,3′-diaminobenzidine tetrahydrochloride (DAB) method (ZLI-9017, ZSGQ-BIO, China). Lastly sections were counterstained with Mayer hematoxylin and mounted for microscopic visualization. Following primary antibodies were used: affinity-purified anti-cyclin K [21], vimentin (ZA-051, ZSGQ-BIO), CD117 (ZM-0437, ZSGQ-BIO), Ki-67 (ZM-0166, ZSGQ-BIO).

### Immunofluorescence

Sections were prepared as described in immunohistochemical analysis except without treatment with 3% H_2_O_2_. Samples were incubated with primary antibodies overnight at 4°C, washed in PBS four times, and then incubated with 1∶100 dilution of Cy3 conjugated sheep anti-rabbit antibody (Sigma) or 1∶200 dilution of Alexa Fluor 488 goat anti-mouse IgG (H+L) (Molecular Probes) in 1% saline-buffered BSA for 1 h at room temperature. Following primary antibodies were used: affinity-purified anti-cyclin K, anti-Ki67, anti-CD117 and anti-Plzf (SC-28319, SantaCruz). To visualize DNA, sections were incubated with 1 µg/ml of DAPI. After washing in PBS, coverslips were mounted with Aqueous Mounting Medium (F4680, Sigma), and visualized by Nikon Eclipse Ti-U fluorescence microscope.

To visualize the localization of cyclin K and vimentin simultaneously, sections were incubated with anti-vimentin antibodies first followed by immunofluorescence detection. Both primary and secondary antibodies were then removed by incubation with 10 mM citrate buffer (pH 6.0) in a pressure cooker for 2 min. Cyclin K was then detected by immunohistochemistry. The reason for this modified method is because both primary antibodies are generated from rabbit, and therefore not suitable for the double-staining procedure.

## Results

### Cyclin K is highly expressed in murine testes

**Figure 1:**
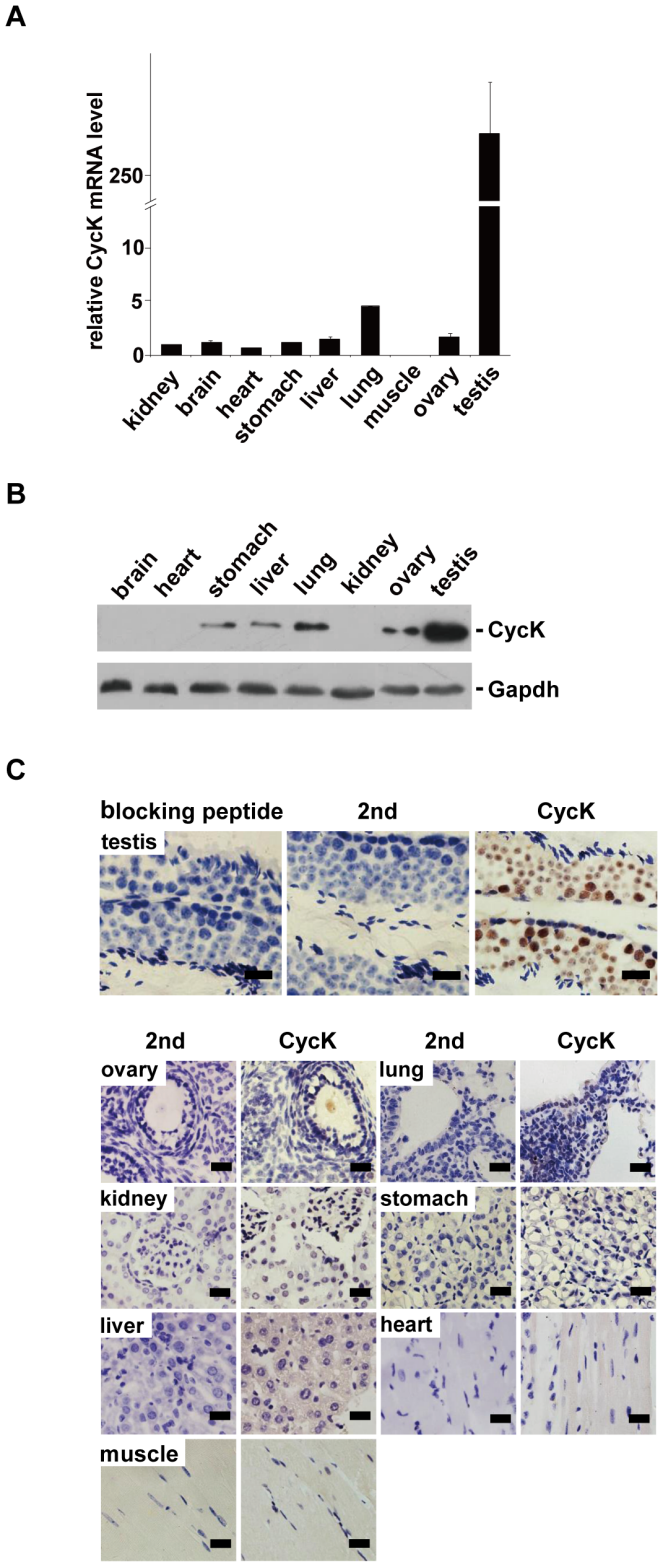
Cyclin K is highly expressed in murine testes. A) The level of CycK mRNA was determined by qPCR. Expression of CycK was normalized to that of Gapdh in three independent experiments. CycK expression in stomach was arbitrarily assigned to be one, and expression levels in other tissues were normalized to that of stomach. Error bars represent SD. B) Protein blot analysis of CycK expression in different tissues. Total tissue lysates were separated by SDS-PAGE and probed by the indicated antibodies. Representative blot of three independent experiments was shown. C) Tissue sections from 2-month-old mice were stained by IHC with anti-CycK antibodies followed by staining with hematoxylin. Upper panel, detection of CycK in testes. Preincubation with epitope peptides eliminated the signal (left). Secondary antibodies alone did not generate any signal (middle). Cyck expression could be seen in different cell types in seminiferous tubules (right). Lower panel,CycK expression in various tissues. In each case, second antibodies alone (2nd) did not produce detectable signals. Of note, staining was carried out on the same day to allow semi-quantitative comparisons of CycK expression levels. Representative results from four independent experiments were shown. Scale bar: 20 µm.

The expression of cyclin K (CycK) was examined under physiological conditions. Total RNA was extracted from different tissues of 2-month-old mice, and the amount of CycK mRNA was quantified by qPCR analyses. As shown in Figure 1A, expression was more than 100-fold higher in testes than that in other organs. Western blot analyses confirmed higher level of CycK protein in testes and weak but detectable expression in the stomach, liver, lung and ovary. Expressions in other organs were hardly detectable (Figure 1B). Of note, using other housekeeping genes including actin and tubulin as normalization standards generated similar results (data not shown).

The expression of CycK was further examined by immunohistochemical staining (IHC). A previously characterized anti-CycK antibody was used [21], and its specificity was further examined under IHC conditions. CycK expression could be easily detected in several cell types within the seminiferous tubules in testes while preincubation with epitope peptides completely abolished the signal. In addition, secondary antibodies alone did not generate any signal (Figure 1C). These results demonstrated that this antibody is highly specific. Tissue sections were also stained with hematoxylin after IHC to visualize different cell types. CycK expression was further examined in other organs by IHC. To ensure semi-quantitative comparison among different tissues, IHC was carried out on the same day. Consistent with qPCR and western blot analyses, IHC revealed the strongest expression of CycK in testes. Same results were obtained reproducibly from different mice (data not shown). Taken together, these results demonstrate that CycK is highly expressed in testes.

### Expression of cyclin K is developmentally regulated during spermatogenesis

**Figure 2:**
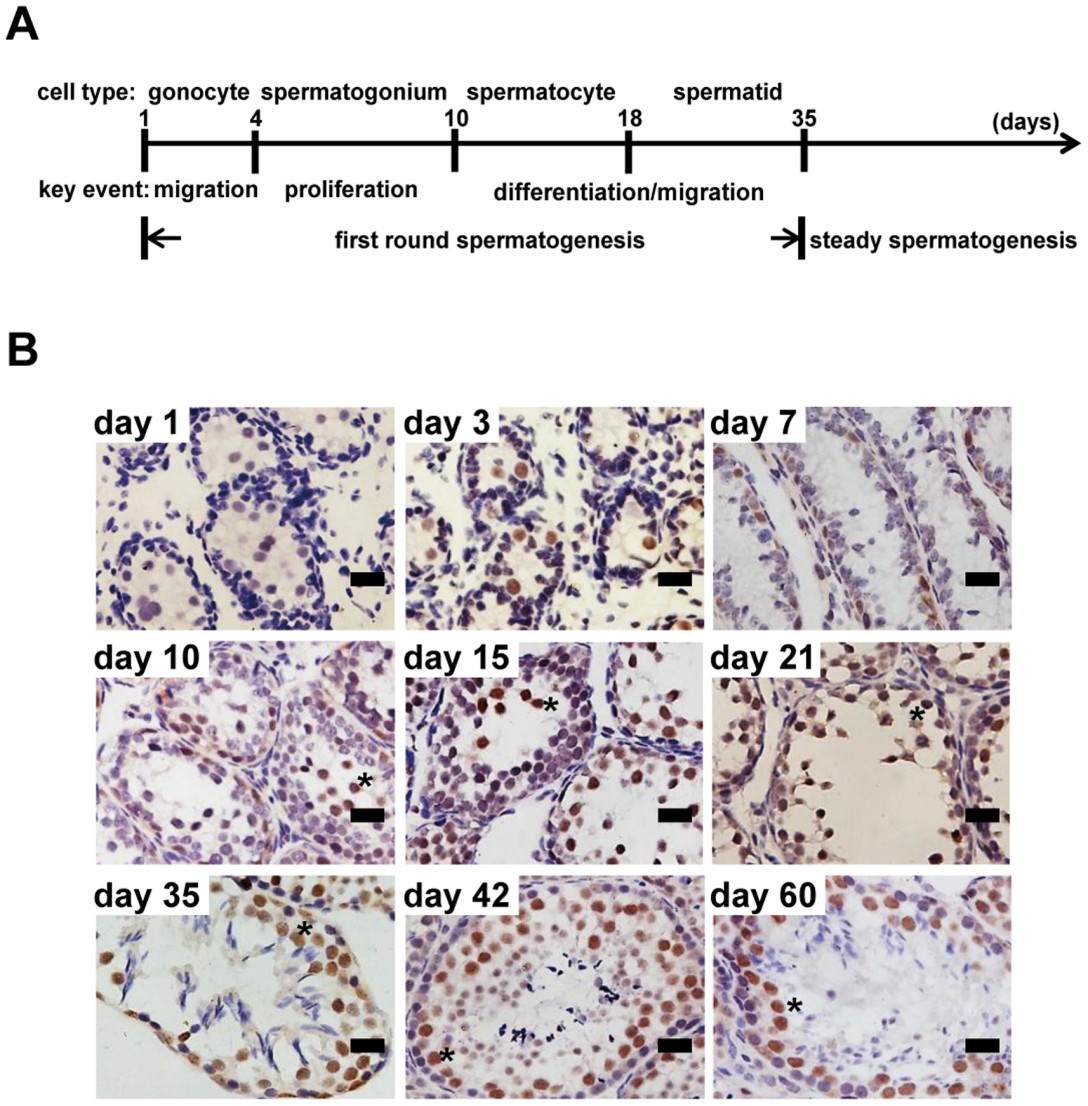
Expression of cyclin K is developmentally regulated during spermatogenesis. A) Schematic presentation of cell types and key events during neonatal and adult spermatogenesis in mice. B) Expression of CycK detected by IHC at different stages of spermatogenesis. Representative results from four independent experiments were shown. Asterisks denote spermatocytes. Scale bar: 20 µm.

The expression of CycK was further examined in testes. We took advantage of the fact that different types of germ cells can be easily recognized during the first round of spermatogenesis in the neonatal period (Figure 2A). To ensure semi-quantitative comparison among different developmental stages, tissue samples from different developmental ages were examined on the same slide (Figure 2B). At day 1 (right after mice were born), CycK was barely detectable in gonocytes (spherical cells near the center of seminiferous tubules and also the biggest cells at this developmental stage). When gonocytes migrate towards the basal membrane, CycK expression was easily detectable (day 3). The expression persisted when gonocytes proliferate and start to transform into spermatogonia (day 7 and day 10, note that there are more CycK-positive cells per seminiferous tubule compared to previous days). When spermatogonia differentiate into spermatocytes (day 15) and spermatids (day 21), CycK expression was most prominent in spermatocytes (round cells not attached to the basal membrane and also the biggest germ cells), and persisted upon completion of first round of spermatogenesis (day 35). The strongest expression was also observed in spermatocytes in steady spermatogenesis (day 42 and day 60). Interestingly CycK expression was notably absent in spermatozoa (day 35, day 42 and day 60).

### Cyclin K is highly expressed in germ cell compartment

**Figure 3:**
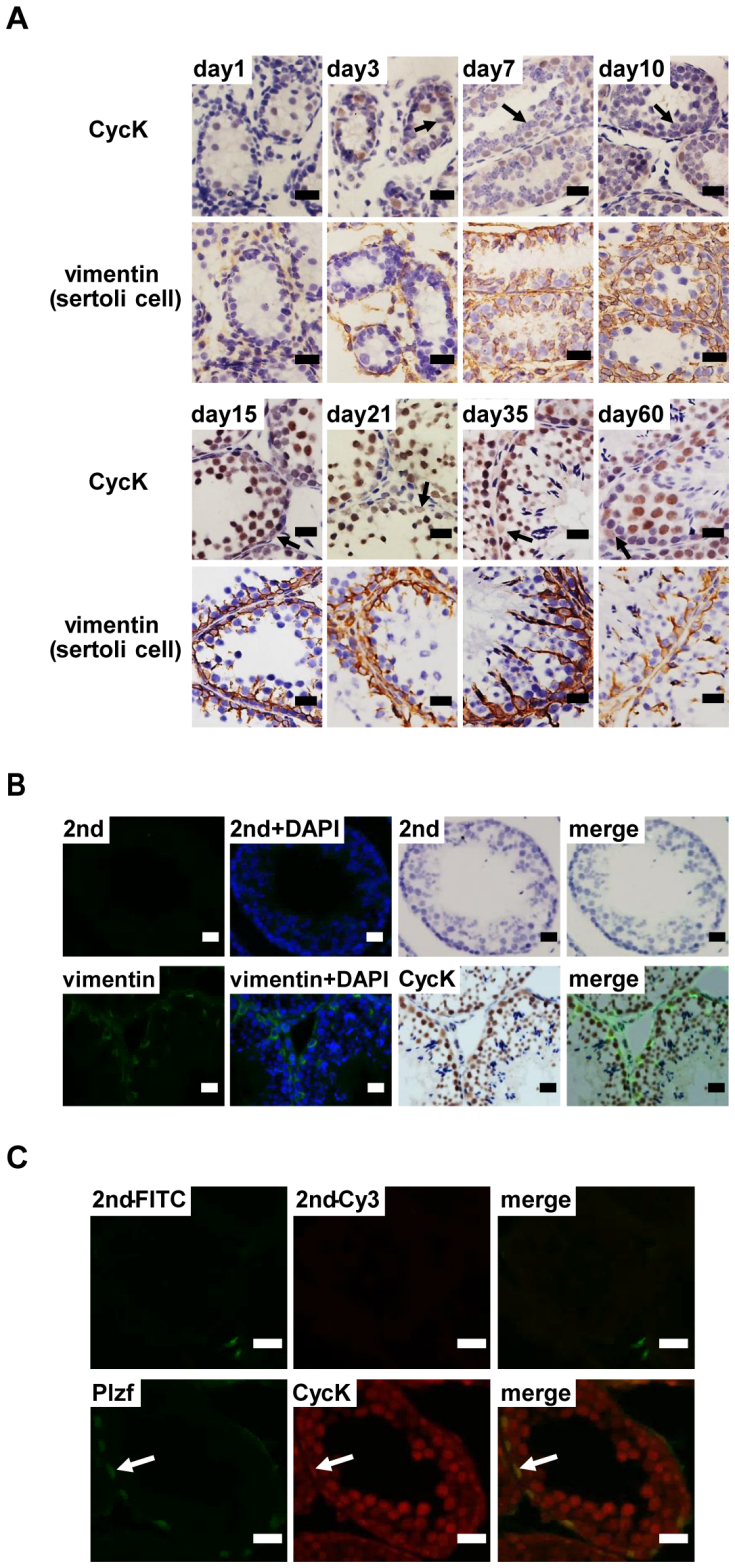
Cyclin K is highly expressed in germ cell compartment. A) Expression of CycK and vimentin at different stages of spermatogenesis. Vimentin is a marker for Sertoli cells. Representative results from two independent experiments were shown. Arrows denote Sertoli cells. B) Detection of CycK and vimentin on the same region of seminiferous tubules. Vimentin was detected by immunofluorescence, followed by detection of CycK by IHC. DNA was visualized by DAPI staining. C) Expression of CycK could be detected in Plzf-positive spermatogonial stem cells. Scale bar: 20 µm.

The expression of CycK was also examined in Sertoli cells, the main somatic cell type that constitutes the microenvironment for spermatogenesis, at different development stages. Sertoli cells can be easily identified by the presence of prominent nucleoli [28]. Immature Sertoli cells did not show detectable CycK expression by IHC (Figure 3A, day 1 and day 3). Consistent with previous reports, the number of immature Sertoli cells increased markedly before day 21 (round cells with prominent nucleoli and perinuclear staining of vimentin). CycK expression could not be detected in these cells (Figure 3A, day 7 and 10). However, weak expression of CycK could be detected in mature Sertoli cells, marked by extended cytoplasmic expression of vimentin from day 35 and beyond (Figure 3A). Double staining of CycK and vimentin further confirmed that CycK is highly expressed in vimentin-negative germ cell compartment (Figure 3B). Finally co-localization studies by immunofluorescence confirmed presence of CycK in spermatogonial stem cells marked by Plzf expression (Figure 3C) [26], [27]

Taken together, the expression of CycK is developmentally regulated during spermatogenesis. During neonatal spermatogenesis (from dpp1 to dpp 35), CycK is highly expressed in gonocytes and undifferentiated spermatogonia but not Sertoli cells. During steady spermatogenesis (from dpp35 and beyond), CycK is prominently expressed in SSCs as well as primary spermatocytes, and weakly expressed in early stage of spermatids as well as Sertoli cells. No expression can be detected in late stage of spermatids, spermatozoa, Leydig cells or myoid cells (Table 1).

**Table 1:** Differential expression of cyclin K during postnatal testis development.

Cell Type	day 1	day 3	day 7	day 10	day 15	day 21	day 35
gonocytes	−/+	+++	N/A				
undiff. permatogonia	N/A		++	++	++	++	++
diff. spermatogonia	N/A		−	−	−	−	−
spermatocytes	N/A			−/+	+++	+++	+++
round spermatids	N/A					+	+
elongated spermatids	N/A					−	
spermatozoa	N/A						−
sertoli cells	−	−	−/+	−/+	+	+	+

Footnote: N/A: not applicable. −: no expression. −/+: weak or no expression. +: detectable expression. ++: strong expression. +++: strongest expression.

### Expression of Cyclin K in human tissues

**Figure 4:**
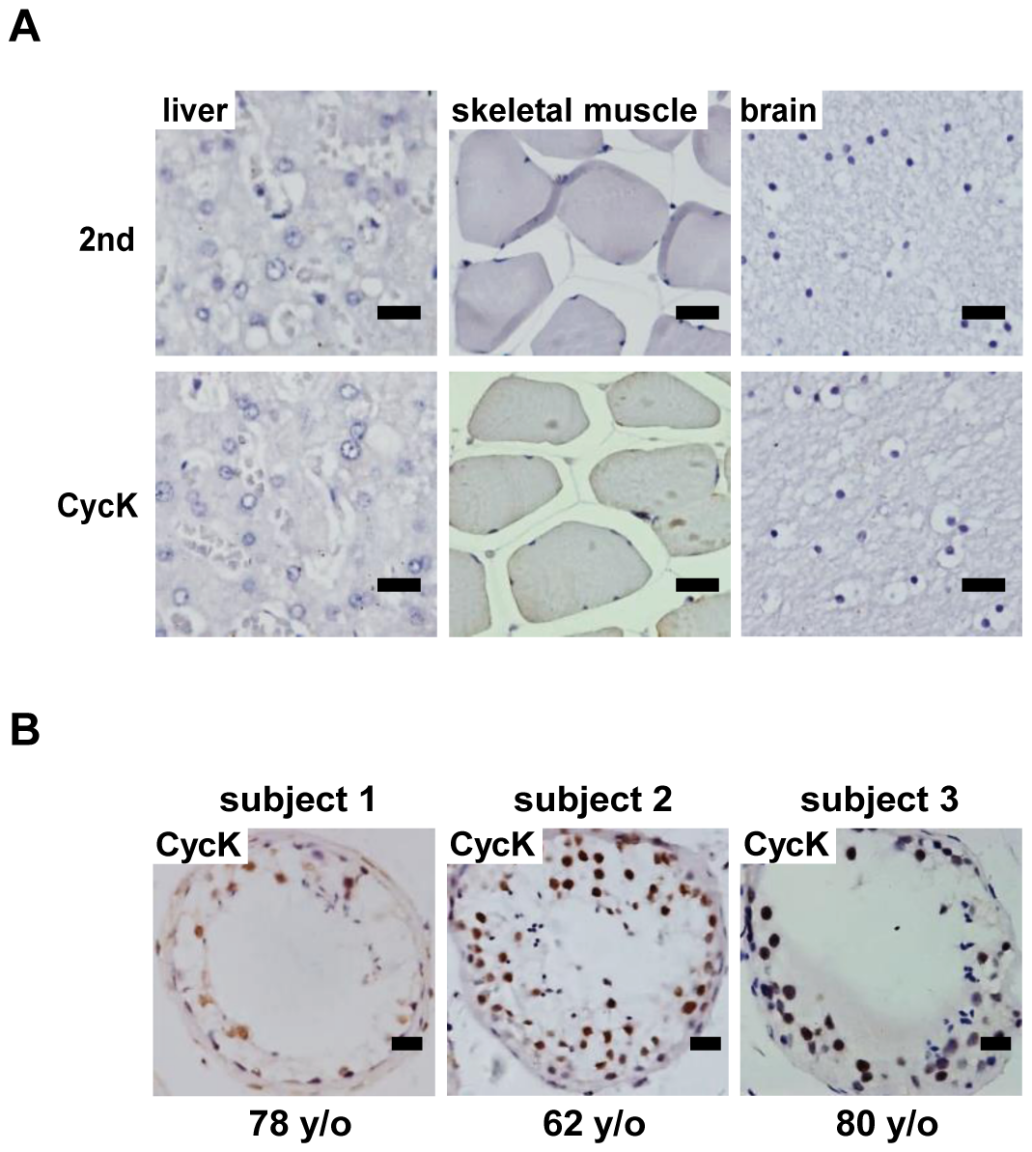
Expression of cyclin K in human tissues. A) Expression of CycK in liver, brain and muscle tissues of an 18-year-old male. B) Expression of CycK in testes of three individual with different ages. Scale bar: 20 µm.

**Figure 5:**
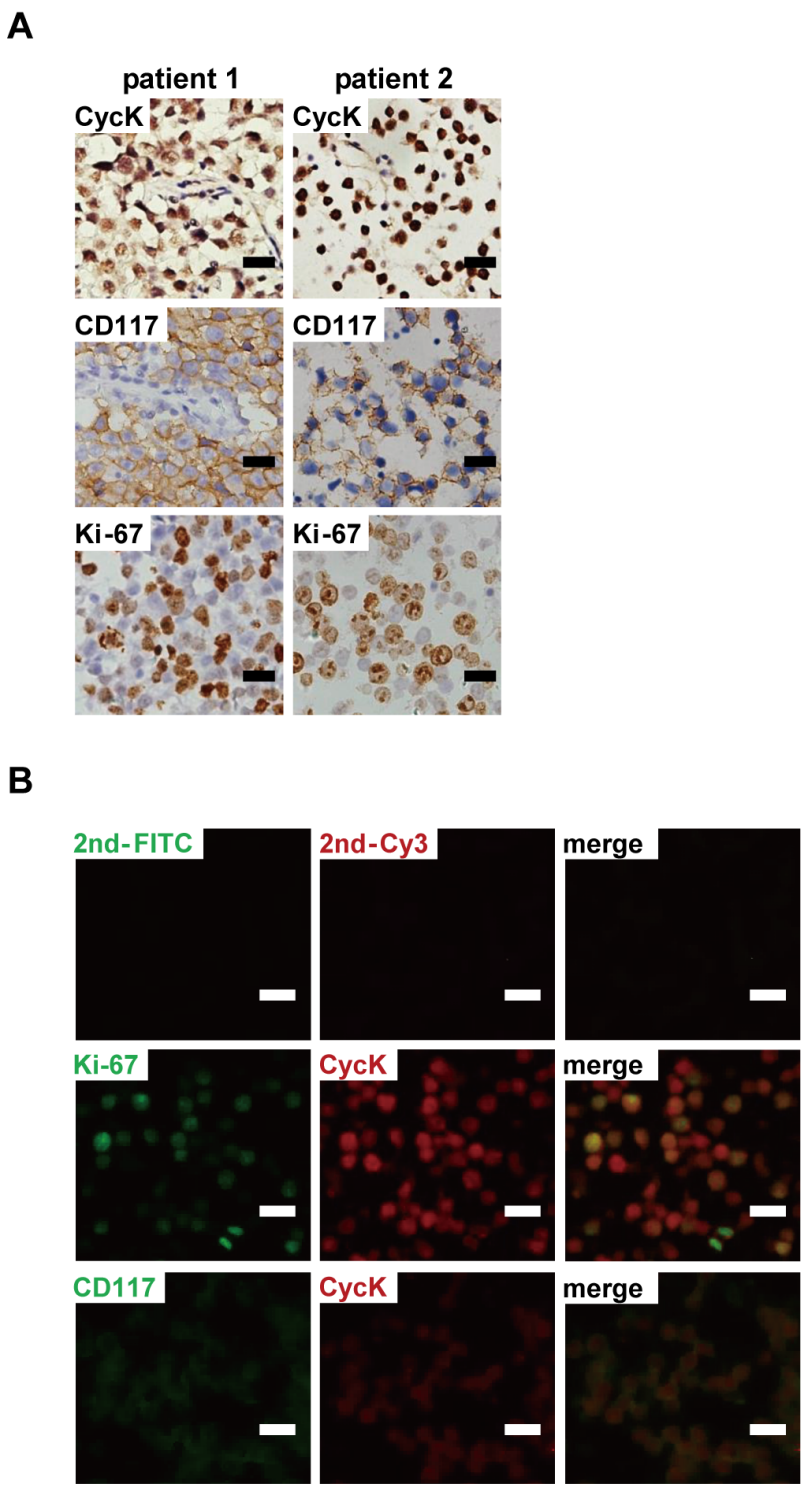
Cyclin K is highly expressed in human testicular cancers. A) High level of CycK expression was detected in human seminomas. B) CycK expression was largely co-localized with Ki-67 and CD117, markers of proliferation and seminomas, respectively. Scale bar: 20 µm.

The expression of CycK was investigated in human tissues by IHC. As shown in Figure 4A, no CycK expression could be detected in brain, liver and muscle tissues obtained from a healthy, 18-year-old Chinese Han male. In contrast, CycK expression could be easily detected in spermatocytes in testis biopsies from three Chinese Han individuals of different ages (Figure 5B).

### Cyclin K is highly expressed in human testicular tumors

The expression of CycK was further investigated in human testicular tumors. As expected, CD117+ seminomas showed robust mitotic activities, indicated by prevalent Ki-67 staining [29]. Interestingly CycK was also highly expressed in cancerous cells but low in surrounding normal cells in all three seminoma cases (two examples were shown in Figure 5A). Immunofluorescence studies revealed that CycK expression was largely localized in CD117+ and Ki-67+ cells (Figure 5B).

### Cyclin K is required for proliferation of testicular cancer cells

**Figure 6:**
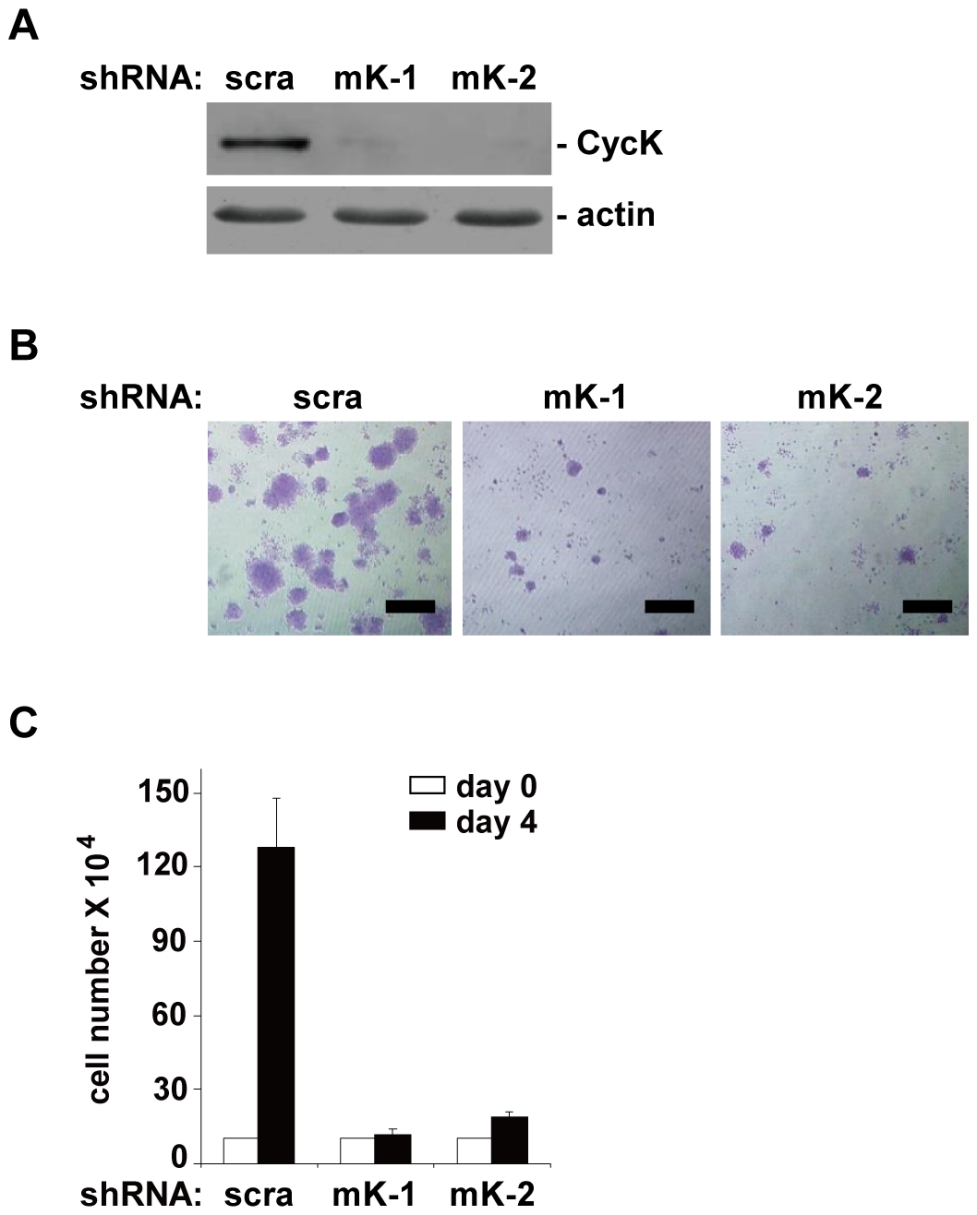
Cyclin K is required for proliferation of testicular cancer cells. A) Knockdown of CycK by either scrambled (scr) or two different shRNA constructs (mK-1 and mK-2). Cells were grown for four days after knockdown, and collected for protein blot analyses. B) Crystal violet staining of cells four days after knockdown. C) Quantitation of cells in B) from three independent experiments. Error bars represent SD. Scale bar: 500 µm.

Lastly we investigated whether CycK is functionally important in testicular cancer cells in vitro. F9 cells, originally derived from testicular teratomas, were used. Two different shRNA constructs targeting CycK were generated (Figure 6A). F9 cells were transduced with individual shRNA constructs, and were grown for four days. Cells transduced with scrambled shRNA (scra) proliferated at a roughly exponential rate. In contrast, when CycK was knocked down, cell proliferation essentially ceased (Figure 6B and 6C).

## Discussion

The functions of cyclin K (CycK) are not well understood. Although it was previously thought that it interacts with CDK9 to regulate transcription elongation, recent findings from several labs have established that CycK interacts with CDK12 and CDK13 but not CDK9 [17], [20], [21]. Similar to CycK, the functions of CDK12 and CDK13 are not well characterized either. CycK, CDK12 and CDK13 are localized in nuclear speckles, indicating that they may be involved in some aspects of transcription [24], [30]. Consistent with this notion, their immunoprecipitates can phosphorylate the carboxyl terminal domain (CTD) of the largest subunit of RNA polymerase II [31], [32], and Drosophila CDK12 is found to be present on the transcribed regions of active genes [22]. In this study, we demonstrate that CycK expression is highly regulated during spermatogenesis. It will be of interest to generate anti-CDK12 and CDK13 antibodies to determine the distributions of CDK12 and CDK13. Knockout studies, perhaps conditional knockout in testes, will also be instructive to elucidate the functions of these novel proteins.

Previously we have shown that cyclin K is highly expressed in ESCs, and is required to maintain pluripotency. Many genes highly expressed in ESCs are also expressed in germ cells, and are functionally important for germ cell development at different stages. Recent studies have established core pluripotency transcription factors bind similar regions across the genome, and maintain ESCs self-renewal and pluripotency in a collaborative manner. However, these genes seem to express at different stages of germ cell development. Notably, Oct4 and Sox2 are present in SSCs but absent in spermatocytes while Nanog shows the opposite expression pattern. Interestingly Oct4 and Sox2 are needed to acquire and maintain pluripotency when somatic cells are induced to convert into ESC-like iPS cells [33], [34], while Nanog is only required for initiation but not maintenance of pluripotency [13]. Notably ESCs and iPS cells generate teratomas efficiently in testes [2]. It is tempting to speculate that one of the reasons for these core pluripotency factors to express at the different developmental stages of germ cells is to acquire pluripotency and to avoid tumorigenesis at the same time. In present study, we demonstrate that the expression of CycK is highly expressed in both SSCs and spermatocytes. It will be of interest to determine whether CycK fits in the regulatory network of Oct4, Sox2 and Nanog or presents a separate cellular pathway.
